# Development of Sustainable Biocatalytic Furfurylamine Production in a Magnetic Field-Assisted Microfluidic Reactor

**DOI:** 10.1021/acssuschemeng.5c04752

**Published:** 2025-09-11

**Authors:** Marko Božinović, Marjan Jereb, Borut Šketa, Aljaž Gaber, Mojca Seručnik, Janez Košmrlj, Polona Žnidaršič-Plazl

**Affiliations:** Faculty of Chemistry and Chemical Technology, 112794 University of Ljubljana, Večna pot 113, Ljubljana 1000, Slovenia

**Keywords:** furfural, furfurylamine, ω-transaminase, biomass valorization, magnetite nanoparticles, enzyme immobilization, green chemistry

## Abstract

The increasing demand for furfurylamine (FA), a versatile biobased building block, necessitates the development of efficient and sustainable production processes. This study presents a continuous biocatalytic process for the amination of furfural (FUR) to FA, aligning with green chemistry principles and circular economy strategies. A systematic screening of ω-transaminases (ω-TAs) and amine donors identified *N*-His_6_-ATA-wt and (*S*)-(−)-α-methylbenzylamine as the optimal pair, achieving a 96% FA gross yield within 30 min at equimolar substrate concentrations, surpassing previously reported ω-TA-based FA productions. To enable biocatalyst long-term use in continuous processes, the enzyme was covalently immobilized on synthesized and functionalized magnetite nanoparticles (MNPs) using glutaraldehyde (GA) as a cross-linker. At optimized immobilization conditions, 92.8% recovered activity was achieved with 80 mg enzyme/g dry MNPs and 2% (v/v) GA in a batch process. The immobilized biocatalyst was integrated into a custom 3D-printed magnetic field-assisted microreactor and evaluated in continuous-flow operation for 18 days. The system reached a maximum space-time yield of 1.07 g/(L h) and a total turnover number of 2.04 × 10^7^. These results, along with favorable green chemistry metrics, highlight the potential of this integrated approach—combining enzyme engineering, nanomaterials, and flow technology—for scalable and sustainable FA production.

## Graphical abstract

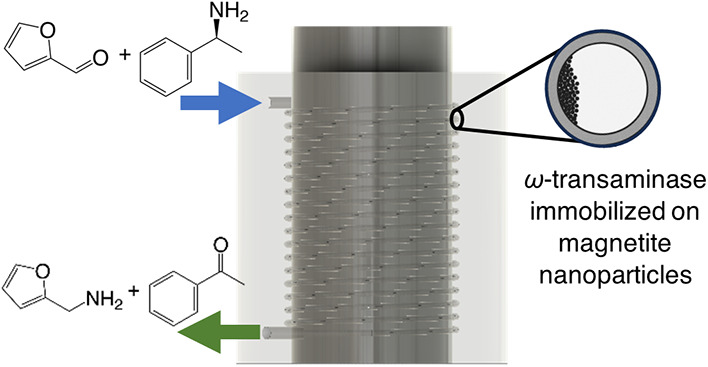

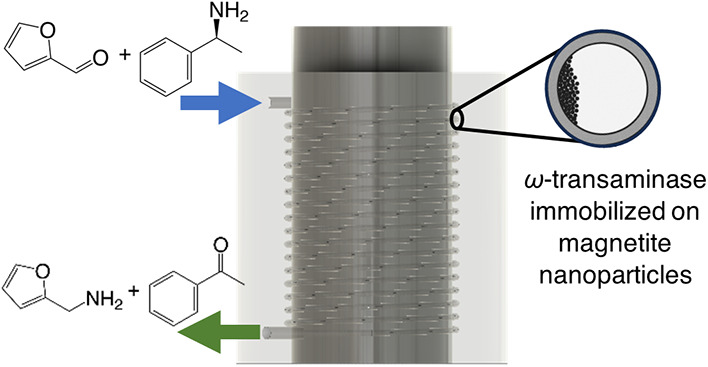

## Introduction

Furfurylamine (FA) has attracted considerable attention due to its numerous current and potential industrial applications, e.g., as an intermediate in the production of pharmaceutical compounds such as antiseptics, antihypertensives, and diuretics.^1^ Selective reductive amination of furfural (FUR) is a reliable process to produce FA, in which FUR and ammonia are mixed with poly­(*N*-vinyl-2-pyrrolidone)-capped Ru-supported hydroxyapatite (Ru-PVP/HAP) and H_2_ (≥2.5 bar). However, the conventional approach for this synthetic reaction has some drawbacks. The reductive conditions pose a challenge as they render the furan ring sensitive, leading to the formation of undesired byproducts, often in the form of secondary or tertiary amines. The environmental footprint of this traditional synthesis is further exacerbated by the use of reducing agents, the generation of byproducts, and the high energy consumption.^1,2^ To overcome these drawbacks, considerable efforts have been devoted to developing a more environmentally friendly process for the production of organic amines. In particular, the establishment of a biocatalytic route using ω-transaminases (ω-TAs) is very promising in this context.^3–7^

ω-TAs have also attracted attention for the amination of FUR and its analogs.^1,7,8^ Among the amine donors for ω-TA-catalyzed reactions, isopropylamine (IPA) is preferred in industry because it is inexpensive, is achiral, and generates acetone as a byproduct that can be easily removed from the reaction due to its low boiling point.^9^ d- or l-Alanine as amine donors are also used, but are expensive,^7^ while methylbenzylamine is cheap and often used to produce pure enantiomers.^10^ However, for all the biocatalyzed FUR aminations listed above, a large excess of amine donors limits the practical application of such production processes.

Microreactors offer a transformative approach to bioprocessing by overcoming the challenges associated with traditional batch processes. Their small scale and large surface area-to-volume ratio allow precise control of reaction parameters, such as temperature, pressure, and flow rates, resulting in improved reaction efficiency and product yield. The continuous flow of microreactors facilitates real-time process monitoring and control, reducing the risk of side reactions and improving product quality. All this makes the microreactor ideal for complex biochemical conversions, especially in pharmaceutical and fine chemical synthesis, where precision and efficiency are crucial.^11^ Their miniaturized design reduces the consumption of reagents and solvents, resulting in less waste generation and lower energy requirements, in line with the principles of green chemistry. In addition, the scalability of microreactor systems by numbering up enables a seamless transition from laboratory scale to industrial applications, bridging the gap between research and commercialization.^12^

Enzyme immobilization is an important strategy in biocatalysis, allowing for improved enzyme stability, reusability, and operational control. Immobilization of enzymes on a solid support minimizes enzyme loss during reactions and enables continuous processing. This approach increases the enzymes’ resistance to denaturation and harsh operating conditions and is therefore essential for biotechnological processes in the fields of pharmaceuticals, fine chemicals, and biofuels.^13^ Immobilization of enzymes on magnetite (Fe_3_O_4_) nanoparticles (MNPs) offers unique advantages due to the large surface area of nanoparticles, their biocompatibility, and their magnetic properties. These features facilitate efficient enzyme loading, increase catalytic activity, and allow easy separation from reaction mixtures by external magnetic fields. Additionally, MNPs provide a versatile platform for functionalization, enabling tailored interactions between the enzyme and the support, which further improves the stability and activity of enzyme. This method is particularly valuable in continuous-flow systems, where the rapid separation and reuse of enzymes significantly reduce operating costs and waste generation.^14–16^

Efficient enzyme immobilization, along with continuous operation and process miniaturization, paves the way for the intensification of biocatalytic processes.^11^ This concept refers to a suite of technologies that replace large, energy-intensive, and costly equipment with smaller, more efficient, and cost-effective alternatives or that integrate multiple operations into a single device or streamlined process.^17^ In this context, enzyme immobilization holds strong potential to promote the broader industrial adoption of biocatalysis by enhancing catalyst stability, reusability, and compatibility with continuous processes.^18,19^

The primary objective of this work was to identify the optimal combination of ω-TA and amine donor for the efficient synthesis of FA, targeting the lowest feasible amine donor-to-acceptor ratio. To support the development of a continuous, intensified, and sustainable process, an efficient enzyme immobilization strategy was established, and a novel 3D-printed microreactor was designed and fabricated. A systematic screening of various ω-TAs and amine donors, namely, IPA, d-alanine (ALA), and (*S*)-(−)-α-methylbenzylamine (MBA), was conducted for the transamination of FUR to FA. The effects of enzyme concentration and amine donor-to-acceptor molar ratio on FA production were thoroughly investigated. Key green chemistry metrics, such as atom economy, excess reactant factor, reaction mass efficiency, and amine donor cost per reaction, were assessed and compared with literature values to evaluate process economics and environmental performance. Enzyme immobilization on synthesized and surface-functionalized MNPs was optimized regarding the cross-linker (GA) concentration. The transamination reaction system was then implemented in the 3D-printed microreactor, and the operational stability of the immobilized biocatalyst was assessed under continuous-flow operation.

## Experimental Section

### Materials

Furfural (FUR), furfurylamine (FA), d-alanine (ALA), pyridoxal-5′-phosphate (PLP), sodium pyruvate, isopropylamine (IPA), potassium phosphate dibasic, potassium phosphate monobasic, sodium hydroxide, ampicillin, kanamycin, acetophenone (ACP) and (*S*)-(−)-α-methylbenzylamine (MBA), Tris base, iron­(II) sulfate heptahydrate, iron­(III) chloride pentahydrate, glutaraldehyde 50% (GA), (3-aminopropyl)­triethoxysilane (APTES), hydrochloric acid 35%, imidazole, and materials for preparing Luria–Bertani (LB) medium were all purchased from Sigma-Aldrich (St. Louis, Missouri, USA). Disodium hydrogen phosphate dihydrate, isopropanol, and sodium dihydrogen phosphate monohydrate were purchased from Merck (Darmstadt, Germany). *Dpn*I was purchased from Thermo Fisher Scientific (Waltham, Massachusetts, USA). Isopropyl-β-thiogalactopyranoside (IPTG) was purchased from GoldBio (St. Louis, Missouri, USA). A nickel-plated neodymium (NdFeB) cylindrical magnet, grade N45 with axial magnetization, was purchased from Svet Magnetov (Kamnik, Slovenia).

### Biocatalysts

Three different ω-TAs were screened, namely, *N*-His_6_-ATA-wt, pEG 97-TA­(*R*)-AspTerr, and the mutated version pEG 97-TA­(*R*)-AspTerr-T130M-E133F. *N*-His_6_-ATA-wt is a mutant of the wild-type ω-TA (ATA-wt) with a hexahistidine tag (His_6_-tag) at the *N*-terminus.^20^ The original plasmid of ATA-wt was obtained from the metagenomic library of c-LEcta GmbH (Leipzig, Germany) and shows 92% identity with a potential protein from *Pseudomonas mandelii*.^21,22^ A plasmid for pEG 97-TA­(*R*)-AspTerr, which is an ω-TA from *Aspergillus terreus* with His_6_-tags at the *C*-terminus,^23–26^ was a generous gift from W. Kroutil (University of Graz). A version of the latter, namely, pEG 97-TA­(*R*)-AspTerr-T130M-E133F, was prepared as described in the following section. All ω-TAs used were expressed as soluble intracellular proteins in *Escherichia coli* BL21 (DE 3), as described below.

### Mutation of pEG 97-TA­(*R*)-AspTerr

A variant of pEG 97-TA­(*R*)-AspTerr-T130M-E133F was prepared by changing threonine at position 130 to methionine (T130M) and glutamic acid at position 133 to phenylalanine (E133F) as described by Di et al.^8^ Mutations T130M and E133F were introduced with PCR by amplifying the plasmid pEG 97-TA­(*R*)-AspTerr, bearing the wild-type transaminase sequence, using primers, harboring the mutated nucleic acid sequence: TA97-T130M-E133F–F (TGGCATGCGTCCGTTTGATATTGTTAATAATCTGTATATGTTTGTGC) and TA97-T130M-E133F-R (TATCAAACGGACGCATGCCACGAACACCTTTCAG). Agarose gel electrophoresis was used to confirm the correct size of the final product, which was then treated with *Dpn*I to degrade the template and transformed into chemically competent *E. coli* DH5α cells with heat shock. The cells were plated on LB agar plates with ampicillin and incubated overnight. Recombinant plasmids were isolated from subsequent overnight cultures from single colonies. The correct nucleic acid sequence with introduced mutations was confirmed with Sanger sequencing (Eurofins Genomics, Ebersberg, Germany).

### Enzyme Expression, Cell Lysis, and Protein Purification

Enzymes were expressed as soluble intracellular proteins in *E. coli* BL21 (DE 3). Competent *E. coli* BL21 (DE 3) cells (100 μL) were mixed with 2 μL of the plasmid for transformation and incubated on ice for 30 min. The cells were heated at 42 °C for 45 s and then incubated again on ice for another 2 min. LB medium (500 μL) was added to the transformed cells and incubated at 37 °C in the shaker at 180 min^–1^ for 1 h. After the transformation was done, 300 μL of the transformed cells was transferred to 3 mL of LB medium containing 100 mg/L ampicillin for all three used enzymes and incubated at 37 °C in the shaker at 180 rpm overnight. The content was transferred to the fresh LB medium with dissolved 100 mg/L ampicillin or 50 mg/L kanamycin, depending on which plasmid was used, and incubated in a shaker again until OD_600_ using a UV–vis spectrophotometer (UV-2600, Shimadzu, Kyoto, Japan), reaching 0.6. IPTG was added to reach a final concentration of 0.2 mM, and the enzyme expression took place overnight in the thermostated shaker at 18 °C at 180 min^–1^. Cells were harvested by centrifugation at 4000*g* for 10 min and resuspended in 50 mM Tris–HCl buffer, pH 7.4, which contained 0.1 mM PLP and 5 mM imidazole. Cell lysis was acquired by three cycles of sonication of harvested cells kept on the ice to prevent overheating, followed by centrifugation at 20,000*g* and at 4 °C for 30 min.

For enzyme purifications, HisTrap FF (GE Healthcare, Danderyd, Sweden) was used with loading, washing, and elution buffers; 50 mM Tris–HCl with dissolved in 0.1 mM of PLP and 5, 30, and 250 mM imidazole. The enzymes were further transferred to potassium phosphate buffer (100 mM, pH 7.5) containing 0.1 mM PLP using a centrifuge filter unit Amicon Ultra-4 with a nominal molecular weight limit of 10,000 Da (Merck Millipore Ltd., Cork, Ireland). In the final step, the purified enzymes were flash-frozen and kept at −20 °C until further usage.

### Furfural Biotransformation and Evaluation of Enzyme-Specific Activities

If not stated otherwise, batch experiments were performed in closed test tubes containing 10 mM FUR, 10 mM IPA/ALA/MBA as the amine donor, and 0.1 mg/mL purified enzyme. All components were dissolved in potassium phosphate buffer (100 mM, pH 7.5). The reaction was carried out at 30 °C and stirred with a magnetic stirrer at 300 rpm. The blank reaction was also carried out under the same conditions, but without a biocatalyst. Samples were removed after 5 min, the reaction was quenched by diluting 5-fold with potassium phosphate buffer (100 mM, pH 7.5) prewarmed to 100 °C, and the mixture was heated at 100 °C for a further 5 min. The solution was then analyzed as described below. The initial FUR bioamination reaction rate (*v*_0_) was calculated from the linear part of the curve showing molar FA concentrations over time. One unit (U) was defined as the amount of enzyme catalyzing the conversion of 1 μmol of FUR per min.

The gross yield (eq 1) and the space–time yield (STY) (eq 2) were calculated as follows:

$$\mathrm{Gross}\;\mathrm{yield}=\frac{c_{\mathrm{FA}}}{c_{0,\mathrm{FUR}}}\times 100\%$$ 1

$$\mathrm{STY}=\frac{c_{\mathrm{FA}}}{t}$$ 2

where *c*_FA_ is the FA concentration [mM], *c*_0,FUR_ is the FUR initial concentration [mM], and *t* is the time [h].

In some batch biotransformations, the concentrations of IPA, ALA, and MBA were increased to achieve the molar ratios with FUR described in the Results and Discussion section, while all other conditions remained unchanged.

### The Influence of Enzyme Concentration on Furfurylamine Synthesis

The experiments were performed in closed test tubes with *N*-His_6_-ATA-wt and MBA as the amine donor, as described in the previous section, with 10 mM FUR and equimolar MBA concentration. The only change was the enzyme concentration (γ), which varied from 0.1 to 0.5 mg/mL.

### Enzyme Immobilization on Functionalized Magnetite Nanoparticles

#### MNP Synthesis and *In Situ* Modification with APTES

The protocol for the synthesis of MNPs and their functionalization with APTES was adopted from Bôa Morte et al.^29^ Briefly, 2.34 g of FeSO_4_·7 H_2_O and 1.20 g of FeCl_3_·5H_2_O were dissolved in 200 mL of Milli-Q water in a flask under inert atmosphere at 50 °C with constant stirring at 600 rpm for 5 min. 8 M NaOH solution was added until the pH reached 10, and stirring was continued for another 5 min. The temperature was then increased to 80 °C, and the pH was adjusted to 8 with a 4% (v/v) aqueous HCl solution.

For *in situ* modification of MNPs, 88.5 μL of APTES was slowly added to the reaction mixture under constant mixing at 500 rpm. The mixture was stirred at 80 °C for 1 h to complete the surface modification. The particles were washed with isopropanol, Milli-Q water, and 100 mM sodium phosphate buffer (pH 7). After activation, the modified nanoparticles (MNP-APTES) were dried in a vacuum chamber to obtain 2.43 g of a dry powder.

#### Enzyme Immobilization on MNP-APTES via Glutaraldehyde

The MNP-APTES particles were suspended in 100 mM sodium phosphate buffer (pH 7) at a ratio 1:200 (w/v) and activated with GA at the final concentration of 2% (v/v) in the buffer. The mixture was incubated at 25 °C for 1 h. After activation with GA, the particles were successively washed with isopropanol, Milli-Q water, and 100 mM sodium phosphate buffer (pH 7) and then dried in a vacuum chamber to obtain the dry particles (MNP-APTES-GA). To immobilize the enzyme, *N*-His_6_-ATA-wt (0.4 mg/mL, 1 mL) was added to the MNP-APTES-GA particles in 100 mM sodium phosphate buffer (pH 7), with enzyme amounts of 80–100–120 mg per gram of the dry activated MNPs, and incubated for 30 min. The effects of GA concentration on the recovered enzyme activity and immobilization yield were evaluated at 1, 2, and 3% (v/v) of GA and at 80 mg of enzyme per gram of the dry activated MNPs. All other conditions, including the volume of APTES added, the temperature (25 °C), and the buffer (100 mM sodium phosphate buffer, pH 7), were adopted from Bôa Morte et al.^29^ These parameters were not taken into consideration for optimization in the present study.

#### Determination of Enzyme Immobilization Yield and Recovered Activity

The enzymatic activity of free and immobilized enzyme was assessed by monitoring FUR bioamination to FA. Batch experiments were performed in closed Eppendorf tubes, each containing 10 mM MBA, 10 mM FUR, and *N*-His_6_-ATA-wt enzyme immobilized on MNPs. All components were dissolved in 100 mM potassium phosphate buffer (pH 7.5), and the total volume was 1.05 mL. The reaction was carried out at 30 °C with stirring at 1500 rpm for 5 min in a thermoblock and quenched as described above. The immobilization yield was calculated using eq 3, and the recovered activity was determined using eq 4:^13,30^

$$\mathrm{Immobilization}\;\mathrm{yield}=\frac{\mathrm{offered}\;\mathrm{activity}-\mathrm{unbound}\;\mathrm{activity}}{\mathrm{offered}\;\mathrm{activity}}\times 100\%$$ 3

$$\mathrm{Recovered}\;\mathrm{activity}=\frac{\mathrm{observed}\;\mathrm{activity}}{\mathrm{offered}\;\mathrm{activity}}\times 100\%$$ 4

The observed activity refers to the activity of the enzyme immobilized on MNPs, while the offered activity corresponds to the enzyme activity added to the tube. The unbound activity represents the total enzyme activity that did not bind to the MNPs. This unbound activity was measured by attaching magnets to the bottom of the Eppendorf tube, allowing the MNPs to detach, and transferring the supernatant to a new Eppendorf tube to measure the activity as described above. All activities are expressed in units (U), where one U is defined as the amount of enzyme catalyzing the conversion of 1 μmol FUR per min.

#### Microreactor Setup

The microreactor (Figure 1) was designed using CAD software (Fusion 360, Autodesk, California, USA) and microfabricated with an Asiga Pro 4K 45 3D printer (Asiga, Alexandria, Australia). It was printed using FunToDo NanoClear resin (3D Resine, Bruay-sur-l’Escaut, France), with printing parameters set according to the resin manufacturer’s standard recommendations. The microreactor consists of a coil structure with a circular tube having an inner diameter of 0.8 mm wrapped around a cylindrical nickel-plated neodymium permanent magnet (diameter: 2.2 cm, height: 3 cm). The total tube length in the coil was 131.5 cm, resulting in a reactor volume of 661 μL. High-grade polytetrafluoroethylene (PTFE) tubing with an inner diameter of 0.8 mm (Darwin Microfluidics, Paris, France) was used to connect the syringe to the 3D-printed microreactor.

**Figure 1:**
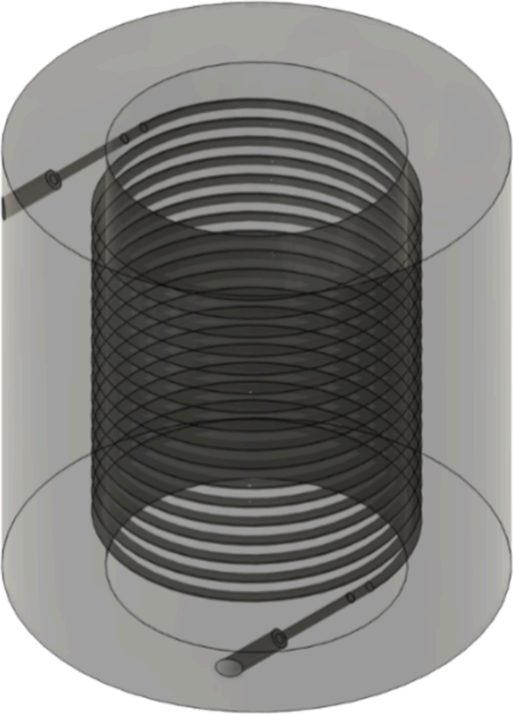
3D fabricated microreactor for FUR bioamination to FA with immobilized *N*-His_6_-ATA-wt on MNP-APTES-GA using an Asiga 3D printer and FunToDo NanoClear resin.

#### Enzyme Immobilization in a Microreactor

The MNP-APTES-GA previously activated in the batch with 2% (v/v) GA were introduced into the reactor in sodium phosphate buffer (100 mM, pH 7). The cylindrical permanent magnet was positioned in the opening to keep the particles within the microreactor. Purified *N*-His_6_-ATA-wt (661 μL, γ = 0.4 mg/mL) in 100 mM sodium phosphate buffer (pH 7) was pumped through the reactor at a flow rate of 5 μL/min using a Harvard syringe pump (Holliston, Massachusetts, USA). After 132.2 min of enzyme introduction, the inlet was switched to a sodium phosphate buffer (100 mM, pH 7), and an additional 661 μL of buffer was passed through the system to wash unbound enzymes from the MNPs.

The immobilization yield (eq 3) and recovered activity (eq 4) were then determined as explained above. The enzyme load was calculated using eq 5, where *V*_reactor_ is the microreactor void volume.

$$\mathrm{Enzyme}\;\mathrm{load}=\frac{\mathrm{offered}\;\mathrm{activity}-\mathrm{unbound}\;\mathrm{activity}}{V_{\mathrm{reactor}}}$$ 5

#### Continuous Transamination in the Microreactor

An equimolar concentration of FUR and MBA (10 mM) in potassium phosphate buffer (100 mM, pH 7.5) was pumped with a syringe pump into a 3D-printed reactor with enzyme immobilized on MNP-APTES-GA at flow rates of 15 to 150 μL/min at room temperature. Once steady-state conditions were reached, samples were taken from the reactor outlet and analyzed by HPLC to determine the concentrations of FUR, MBA, FA, and ACP. Following each run, the reactor was cleaned by flushing with pure isopropanol at a flow rate of 25 μL/min for 1 h using a syringe pump, followed by Milli-Q water using the same conditions. The reactor was then dried with compressed air and reloaded with fresh MNP-APTES-GAs for the subsequent run.

The operational stability of the microreactor system was evaluated by performing continuous biotransformation over several days. The reaction mixture, consisting of an equimolar concentration of FUR and MBA without exogenously added PLP, was pumped through the 3D-printed microreactor at a flow rate of 15 μL/min using a syringe pump. Samples were taken daily from the microreactor outlet and analyzed by HPLC for substrate and product concentration determination. Gross yield was calculated using eq 1, where the FUR concentration in the inlet (*c*_inlet,FUR_) was used instead of *c*_0,FUR_. STY was calculated using eq 2, replacing *t* with residence time (τ).

Operational stability was quantified using relative productivity defined as the ratio of the observed STY on a given day to the initial STY (eq 6):

$$\mathrm{Relative}\;\mathrm{productivity}=\frac{{\mathrm{STY}}_{observed}}{STY_{\mathrm{initial}}}$$ 6

STY was calculated according to eq 2, using the residence time (τ) of 44.07 min, which was the highest τ tested.

The enzyme catalytic constant (*k*_cat_) and the deactivation rate constant (*k*_d_) were determined as described in the Supporting Information (Figures S5 and S6). The total turnover number (TTN) was calculated from estimated *k*_cat_ and *k*_d_ values using eq 7:

$$TTN=\frac{k_{\mathrm{cat}}}{k_{d}}$$ 7

Enzyme leaching during the process was assessed by measuring the enzyme activity in the outlet samples. Each sample was analyzed by HPLC immediately after sampling and then reanalyzed after incubation at room temperature for 2 h. An increase in product concentration during this period would have indicated enzyme leaching from the microreactor.

#### Green Chemistry Metrics and Reaction Economics

Several green chemistry metrics and economic parameters were evaluated for the reactions with various amine donors, including atom economy (eq 8), excess reactant factor (eq 9), and the reaction mass efficiency (eq 10).^27,28^

$$\mathrm{Atom}\;\mathrm{economy}=\frac{\mathrm{molecular}\;\mathrm{mass}\;\mathrm{of}\;\mathrm{FA}}{\mathrm{molecular}\;\mathrm{masses}\;\mathrm{of}\;\mathrm{reactants}}\times 100\%$$ 8

$$\mathrm{Excess}\;\mathrm{reactant}\;\mathrm{factor}=\frac{\mathrm{stoichiometric}\;\mathrm{mass}\;\mathrm{of}\;\mathrm{reactants}+\mathrm{exess}\;\mathrm{mass}\;\mathrm{of}\;\mathrm{reactant}}{\mathrm{stoichiometric}\;\mathrm{mass}\;\mathrm{of}\;\mathrm{reactants}}$$ 9

$$\mathrm{Reaction}\;\mathrm{mass}\;\mathrm{efficiency}=\frac{\mathrm{mass}\;\mathrm{of}\;\mathrm{FA}\;\mathrm{produced}}{\mathrm{total}\;\mathrm{mass}\;\mathrm{of}\;\mathrm{reactants}}\times 100\%$$ 10

To determine the economic efficiency of the FUR amination reactions, the prices of the tested amine donors were sourced from the catalog of Merck KGaA, Darmstadt, Germany (Table S3).

### Analysis

#### HPLC Analysis

HPLC analysis was employed to quantify the concentrations of FUR, FA, MBA, and ACP in the reaction and blank test mixtures. An HPLC system equipped with a diode array detector (Shimadzu, Tokyo, Japan) and a Gemini-NX 3 μm C18 110 Å (150 × 4.60 mm) column (Phenomenex, Torrance, USA) was used to separate and detect the compounds using the isocratic methods described below. The method had a total run time of 7.5 min at a flow rate of 0.8 mL/min.

The mobile phase consisted of acetonitrile and demineralized water (pH adjusted to 11 with 100 mM NaOH) in a ratio of 40:60. The HPLC column was thermostated at 30 °C. FA was monitored by measuring absorbance at 217 nm, while all other compounds were monitored at 250 nm. The retention times were as follows: 2.848 min for FA, 3.364 min for FUR, 4.365 min for MBA, and 6.433 min for ACP. The retention times for the Schiff bases formed were 4.852 min for (*S*,*E*)-1-(furan-2-yl)-*N*-(1-phenylethyl)­methanimine (Schiff base 1), 5.562 min for (*E*)-1-(furan-2-yl)-*N*-isopropylmethanimine (Schiff base 2), 6.492 min for (*R*,*E*)-2-((furan-2-ylmethylene)­amino)­propanoic acid (Schiff base 3), and 6.524 min for (*E*)-1-(furan-2-yl)-*N*-(furan-2-ylmethyl)­methanimine (Schiff base 4).

#### ^1^H NMR Spectroscopic Analysis

An aliquot of 900 μL of the reaction mixture, which was previously centrifuged, was diluted with 100 μL of D_2_O and analyzed by ^1^H NMR spectroscopy (Bruker Avance NEO 600 MHz instrument, Fällanden, Switzerland). The relative distribution of the compounds in the reaction mixtures was determined by integrating the representative resonances.

#### Transmission Electron Microscopy Analysis

For transmission electron microscopy (TEM), the sample was suspended in Milli-Q and homogenized in an ultrasonic bath. The sample was then applied to a TEM grid and dried. The analysis was performed with a TEM (Jeol 2100, Jeol Ltd.) coupled with an energy-dispersive X-ray spectroscope (EDXS, JED 2300 EDS).

#### Vibrating-Sample Magnetometer Analysis

Magnetic characterization was carried out at room temperature using a vibrating-sample magnetometer (VSM) (7307 VSM; Lake Shore Cryotronics, Westerville, Ohio) with a maximum applied field of 10 kOe. All measurements were performed in a continuous-loop mode.

#### Scanning Electron Microscopy Analysis

The sample with MNPs was deposited onto a precoated track-etched membrane with a pore size of 0.2 mm and subsequently dried. Imaging was performed using an Apreo 2 field emission scanning electron microscope (SEM, Thermo Scientific, Waltham, Massachusetts, USA). The analysis was conducted at an accelerating voltage of 2 kV using the T2 Trinity detection system.

## Results and Discussion

### Screening of Enzymes and Amine Donors for Furfural Amination

The first objective of this research was to determine the most cost-effective approach for FA production by identifying the specific ω-TA that catalyzes the reaction of FUR with the selected amine donor, and the minimum amine donor-to-acceptor ratio. A preliminary enzyme screening of three enzymes, namely, pEG 97-TA­(*R*)-AspTerr, its variant pEG 97-TA­(*R*)-AspTerr-T130M-E133F, and *N*-His_6_-ATA-wt at a concentration of γ = 0.1 mg/mL, and three amine donors, namely, ALA, IPA, and MBA, was performed using amine donors and acceptors at equimolar concentrations.

The results obtained with enzyme pEG 97-TA­(*R*)-AspTerr using various amine donors within 3 h were not promising. FA formation was only observed when ALA was used, resulting in 3% FA gross yield with a FUR conversion of 18% (Figure 2a). In contrast, no detectable amount of FA was observed when IPA and MBA were used as amine donors, although FUR conversions of 29 and 28%, respectively, were observed. In all three cases, the conversion of FUR was significantly higher than the corresponding FA gross yield. This can be attributed to the formation of Schiff bases 1, 2, 3, and 4 (Figure S1). When IPA, MBA, and ALA were used as amine donors with the enzyme pEG 97-TA­(*R*)-AspTerr, Schiff bases were formed in all cases (Figure S2).

**Figure 2:**
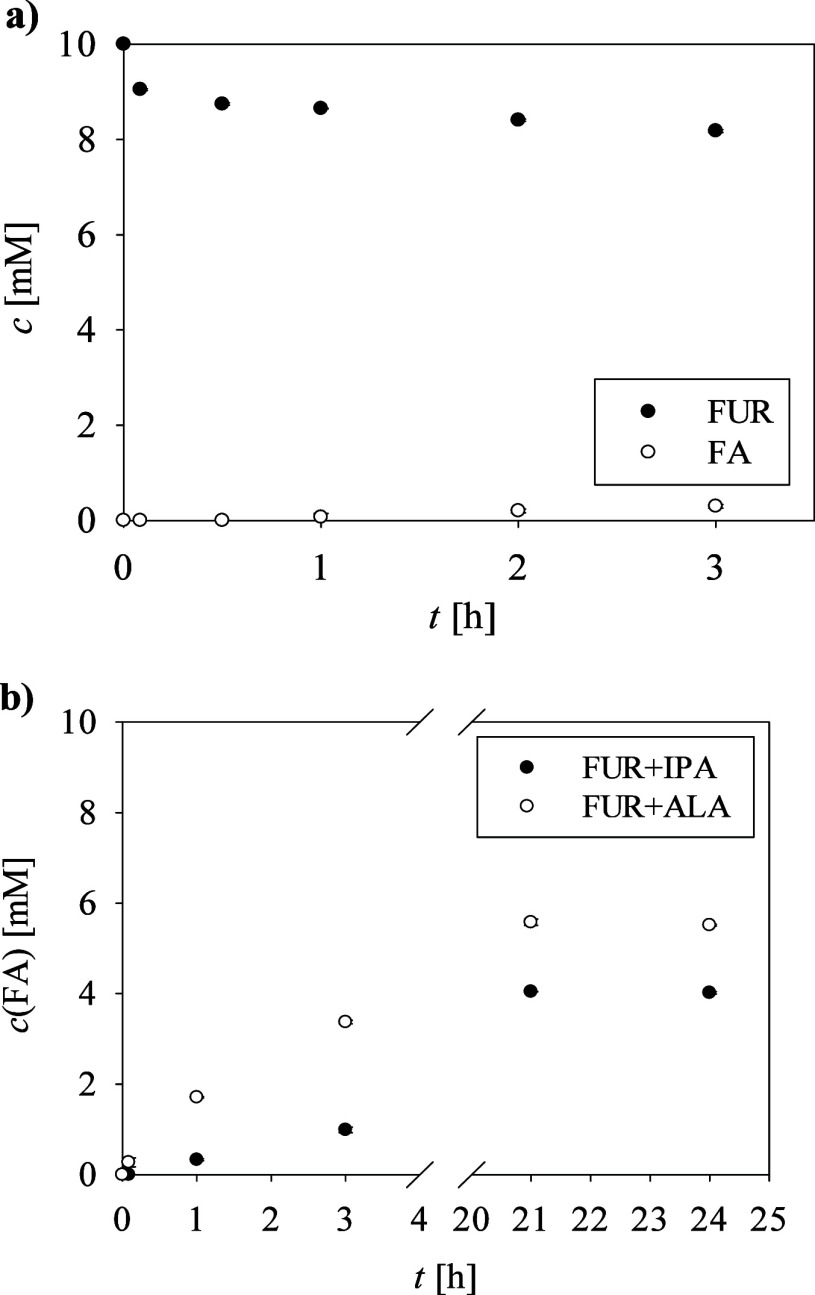
Time dependence of FUR and FA concentrations in the reaction of FUR (10 mM) with pEG 97-TA­(*R*)-AspTerr at γ = 0.1 mg/mL, (a) using ALA in equimolar concentrations and (b) using different amine donors in molar ratios ALA:FUR = 16:1 and IPA:FUR = 10:1. The error bars indicate the standard deviation of triplicate experiments.

Since high amine donor-to-acceptor ratios in enzymatic reactions are known to shift the reaction equilibrium toward FA production,^1,8^ molar ratios of 10:1 for IPA and 16:1 for ALA were tested. This approach resulted in higher gross yields of FA (Figure 2b) compared to the previously used equimolar concentrations. When IPA was used in excess, the gross yield reached 40% after 21 h, while the use of ALA at a molar ratio of 16:1 resulted in a remarkable 55% FA gross yield, which is the most favorable result in terms of the desired product gross yield. Based on eq 2, the STY for the bioamination of FUR using IPA was calculated to be 0.019 g/(L h), while with ALA as an amine donor, it was 0.026 g/(L h). For both enzymatic reactions, the presence of Schiff bases 2, 3, and 4 was confirmed by HPLC (Figure S3). This indicates that the excess amine donor in the reaction mixture with pEG 97-TA­(*R*)-AspTerr shifted the equilibrium toward FA formation but did not prevent spontaneous reactions leading to the formation of Schiff bases.

A 5:1 molar ratio of MBA to 10 mM FUR was also evaluated. However, mixing MBA and FUR at these concentrations in buffer resulted in turbidity, indicating limited substrate solubility in aqueous media. This condition was therefore excluded from further study. To address the same challenge, Zhu et al.^7^ employed a toluene/water biphasic system to dissolve 200 mM FUR, while Di et al.^8^ used a deep eutectic solvent—specifically a choline chloride:malonic acid–water system—to solubilize up to 500 mM FUR.

Subsequent studies focused on testing an engineered ω-TA. Based on the report by Di et al.,^8^ a variant pEG 97-TA­(*R*)-AspTerr-T130M-E133F was constructed to enhance activity toward FUR. The enzyme was tested with all the amine donors mentioned above. No formation of FA was observed with any of the amine donors tested when used in an equimolar ratio with FUR, but the formation of Schiff bases 1, 2, 3, and 4 was observed by HPLC analysis (data not shown). When the amine donor-to-acceptor molar ratios were increased to 10:1 for IPA or 16:1 for ALA, the FA gross yield reached only 1% in 24 h. Compared to the previous cases using the pEG 97-TA­(*R*)-AspTerr enzyme with higher ratios of amine donor to acceptor, this variant did not show similar results and did not shift the reaction equilibrium toward FA production. These results indicate that both *Aspergillus terreus*-derived TAs are ineffective for FA production at equimolar concentrations of the tested amine donors and FUR. On the other hand, with pEG 97-TA­(*R*)-AspTerr, FA formation was observed only at very high donor-to-acceptor ratios. These conditions were not the focus of this study as the use of such an excess of reagents in an industrial setting is hardly economically and environmentally justifiable.

Finally, the enzyme *N*-His_6_-ATA-wt, an (*S*)-selective tetrameric ω-TA from the metagenomic library,^21,22^ was tested with all three amine donors at equimolar concentrations with FUR. When ALA was used as the amine donor, Schiff bases 3 and 4 formed without any trace of FA. With IPA as amine donor, a low gross yield of 1% FA was observed, including the formation of Schiff bases 2 and 4. However, using MBA as the amine donor resulted in a significant 70% gross yield of FA within only 3 h (Figure 3), corresponding to a STY of 0.23 g/(L h). This result represents an 8.85-fold improvement compared to the best result obtained with ALA.

**Figure 3:**
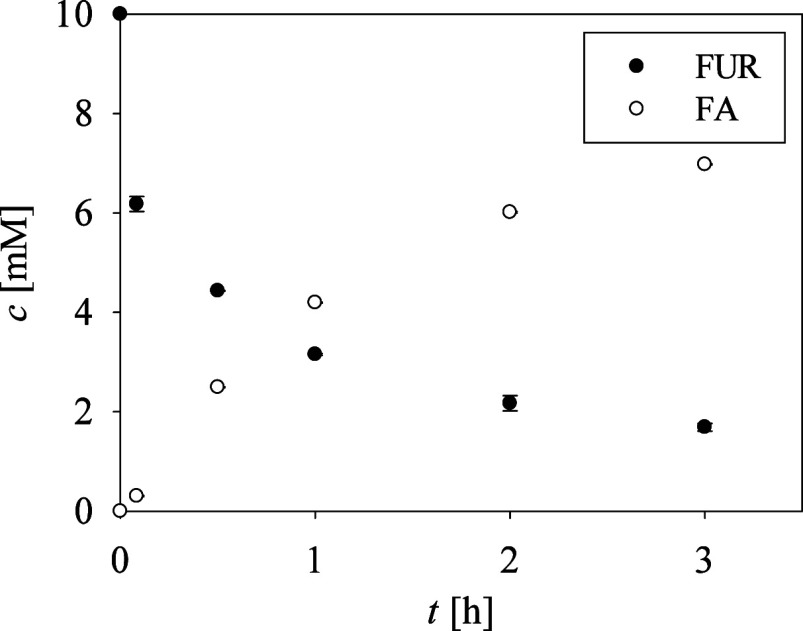
Time dependence of FUR and FA concentrations in the reaction with *N*-His_6_-ATA-wt, employing MBA as an amine donor in equimolar concentrations with FUR; other conditions as indicated in Figure 2. The error bars indicate the standard deviation of triplicate experiments.

### The Effect of Enzyme Concentration on Furfural Biotransformation

The results of enzyme concentration on FA gross yield presented in Figure 4a show that when the enzyme concentration was doubled to γ = 0.2 mg/mL, the gross yield of FA reached 64% in only 50 min. In comparison, this yield was achieved in the previous scenario with the same enzyme at a concentration of 0.1 mg/mL within 3 h, which represents a significant reduction in reaction time. The highest gross yield of 96% was achieved with γ = 0.4 mg/mL at the end of the reaction after 30 min (Figure 4b), with the corresponding STY of 1.87 g/(L h), which is eight times higher than the STY obtained with γ = 0.1 mg/mL. Subsequent increases in the amount of enzyme added showed no further improvements (Figure 4a). Additional confirmation of the reaction’s success was provided by ^1^H NMR spectroscopy, which identified ACP and FA as the main products, with only trace amounts of the starting materials, namely, FUR and MBA. The corresponding ^1^H NMR spectra are available in the HYPERLINK data repository.

**Figure 4:**
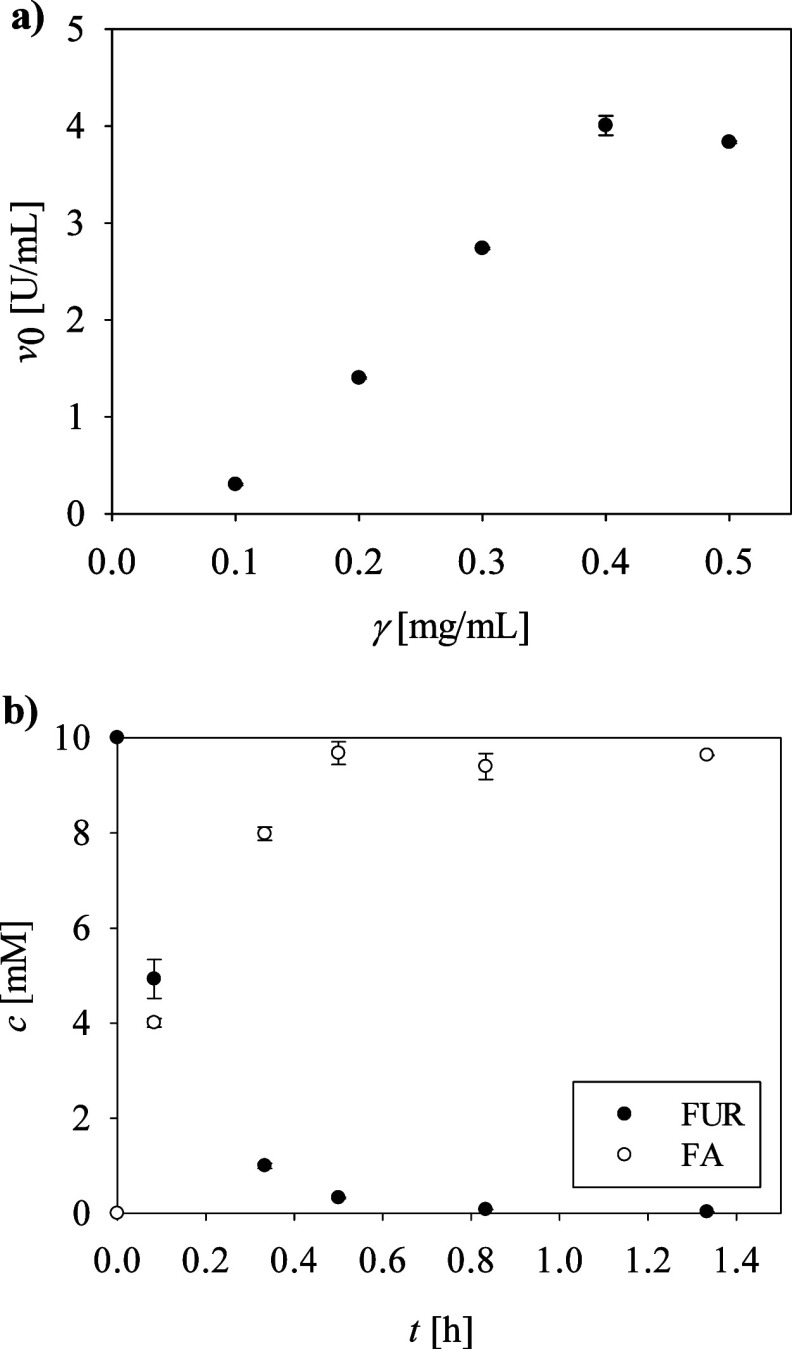
(a) Influence of *N*-His_6_-ATA-wt concentrations (γ) on the initial reaction rate (*v*_0_). (b) Time dependence of FUR and FA concentrations in the reaction with *N*-His_6_-ATA-wt at γ = 0.4 mg/mL using MBA at equimolar concentrations with FUR; other conditions as indicated in Figure 2. The error bars indicate the standard deviation of triplicate experiments.

### Characterization of Synthesized MNPs

The microscopic TEM images revealed that the MNPs obtained are quite polydisperse and have a spherical crystalline morphology (Figure 5a). The core sizes range from 7.0 to 26.7 nm, with an average size of 15.1 ± 3.9 nm. Some particle aggregation was observed, partly due to the drying process of the sample prior to deposition on the TEM grids. Additionally, the APTES coating on the particle surface provides only moderate stabilization, which also contributes to the observed aggregation. Aggregation was also observed using scanning electron microscopy (SEM), as shown in Figure 5b. Although APTES helps to maintain a certain particle dispersion, its stabilizing effect is not optimal. However, this coating is necessary to enable the subsequent immobilization of the enzymes.

**Figure 5:**
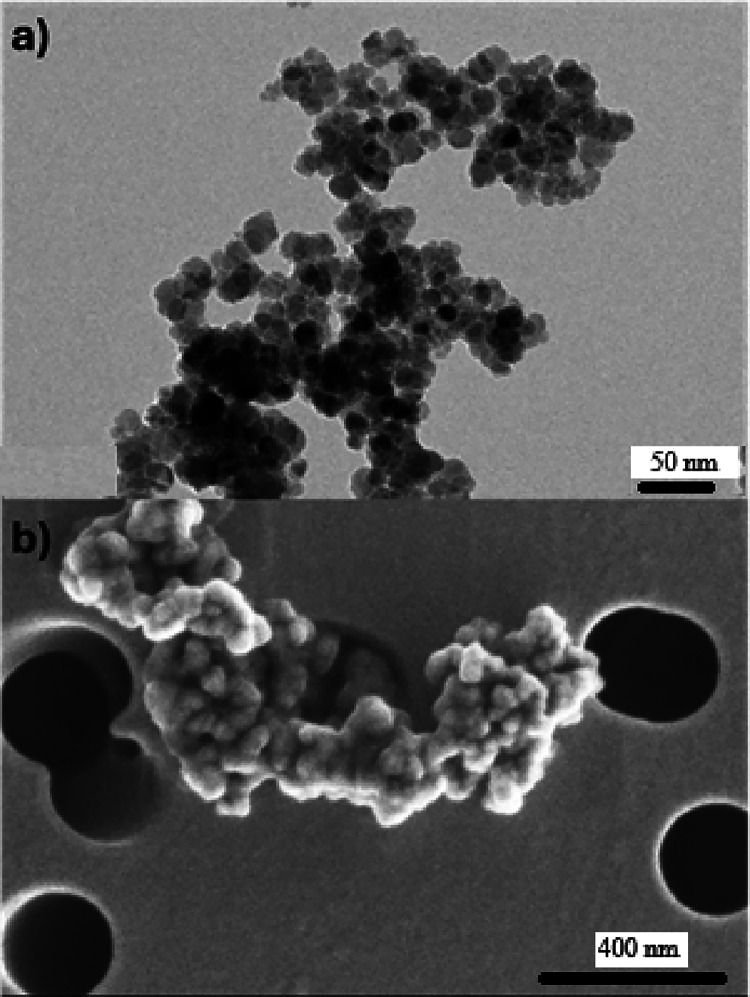
Images of the MNPs taken with (a) TEM and (b) with SEM. In the latter case, the samples are visualized on precoated track-etched membranes with a pore size of 0.2 mm.

The magnetic properties of the synthesized MNPs were investigated using VSM at room temperature. Figure S4 shows the magnetization curve as a function of the applied magnetic field strength. The nanoparticles exhibit ferrimagnetic behavior, as indicated by the hysteresis loop, with a maximum saturation magnetization of 51.497 emu g^–1^, a coercivity of 54.240 Oe, and a remanent magnetization of 4.1372 emu g^–1^.

### Enzyme Immobilization Optimization in the Batch Process

The cross-linker concentration has a significant impact on immobilization efficiency, but its optimal value depends on the specific cross-linker, enzyme, and nanoparticle surface—typically requiring empirical optimization. In this study, GA was used as the cross-linker. GA reacts with primary amine groups on both the nanoparticle surface and the lysine residues of the enzyme, forming Schiff bases.

The effect of the GA concentration was investigated at a fixed enzyme-to-activated MNP mass ratio, while all other immobilization parameters (buffer, pH, and temperature) were adopted from Bôa Morte et al.,^29^ who successfully immobilized *Amano* lipase AK from *Pseudomonas fluorescens* on functionalized MNPs prepared using the same method applied in our work.

Based on recovered activity and immobilization yield (Table S1, Supporting Information), an enzyme loading of 80 mg per gram of dry MNPs was selected for further evaluation of GA concentration, tested at 1, 2, and 3% (v/v). As shown in Table S2 (Supporting Information), the highest recovered activity was achieved at 2% (v/v) GA, which also resulted in the second-highest immobilization yield. Consequently, this concentration was selected for enzyme immobilization in the microreactor. This outcome is consistent with previous reports indicating that insufficient GA can lead to poor immobilization efficiency, whereas excessive GA may impair enzyme activity and stability due to overcross-linking and conformational rigidity.

### Continuous Furfurylamine Production in a Microreactor

The stability of a biocatalyst is crucial for its industrial application, as it reduces process costs and minimizes downtime associated with catalyst replacement. To evaluate the stability of the enzyme and its operating time in a microreactor system, a continuous bioamination of FUR at room temperature without the addition of PLP was performed. As previously described, the exogenous addition of PLP is not necessary for *N*-His_6_-ATA-wt, as this tetrameric transaminase forms a stable complex with PLP.^20–22,31^

MNPs were first activated with 2% (v/v) GA in a beaker and subsequently introduced into the microreactor. A cylindrical permanent magnet was placed in designated slots of the reactor to retain the particles in the microreactor via the magnetic field. Subsequently, the enzyme solution in buffer was introduced using a syringe pump. The resulting enzyme load (eq 5) was 0.39 U/mL, with an immobilization yield of 65% (eq 3) and a recovered activity of 60% (eq 4).

The lower immobilization yield compared to the batch process is likely due to a reduced effective surface area of the functionalized MNPs’ available for enzyme attachment within the microreactor. Under the influence of the magnetic field, the particles accumulated along the reactor wall adjacent to the magnet, rendering the surface facing the magnet inaccessible for immobilization. This uneven particle distribution ultimately led to decreased enzyme loading and lower recovered activity relative to the batch system.

The continuous reaction in a magnetic field-assisted microreactor with *N*-His_6_-ATA-wt immobilized on functionalized MNPs was monitored by analyzing the concentration of FUR and FA at the reactor outlet at different flow rates and thus residence times (τ). As can be seen in Figure 6a, longer residence times resulted in significantly higher FA gross yields, reaching over 80% at τ = 44 min.

**Figure 6:**
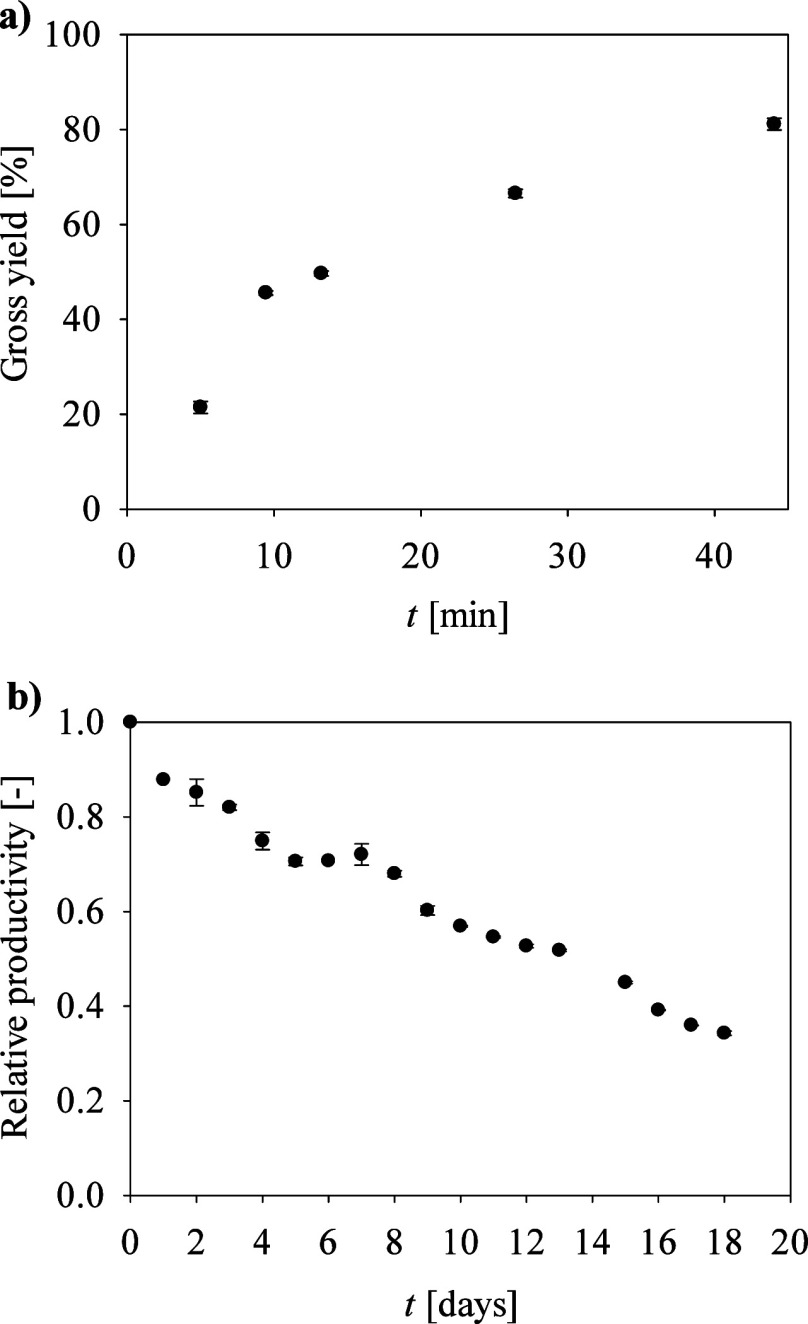
(a) Effect of residence time on FA gross yield in the reaction with *N*-His_6_-ATA-wt immobilized on MNPs; conditions as in Figure 4b. (b) Operational stability of a continuously operated microreactor at τ = 44 min. The error bars indicate the standard deviation of triplicate experiments.

Furthermore, the operational stability of the system was evaluated over 18 days, and the results are shown in Figure 6b. At the beginning of the continuous process, the STY was 1.07 g/(L h). Over time, a decrease in relative productivity (eq 6) was observed, decreasing to 35% by day 18. Analysis of outlet samples performed immediately after collection and after 2 h of samples incubation at room temperature revealed no change in product or substrate concentrations, indicating that the enzyme remained stably immobilized within the microreactor and did not leach from the reactor.

The observed decline in performance is therefore attributed to enzyme deactivation over time, likely caused by acetophenone—a known inhibitor of ω-TAs.^21,22^ As acetophenone exits only through the reactor outlet, the immobilized enzyme is continuously exposed to it along the flow channel, contributing to gradual loss of activity.

The kinetic parameter *k*_cat_ was estimated from batch experimental data fitted using a Ping-Pong Bi–Bi kinetic model without inhibition and was found to be 13 s^–1^ (see the Supporting Information, Figure S5). Additionally, data from the continuous process with immobilized *N*-His_6_-ATA-wt were used to calculate the deactivation rate constant (*k*_d_) based on the first-order deactivation kinetics (see the Supporting Information, Figure S6), yielding a value of 0.0554 day^–1^ and a corresponding enzyme half-life (*t*_1/2_) of 12.51 days. The total turnover number (TTN) for FUR bioamination, calculated according to eq 7, was 2.04 × 10^7^. Comparison with the threshold TTN value considered commercially relevant (>10^6^)^32^ indicates strong commercial potential for industrial application of this process.

To enhance the sustainability of the process, the microreactor was reused across multiple cycles. After each run, the reactor was cleaned with Milli-Q water and isopropanol and then reloaded with fresh MNP-APTES-GAs. This approach enables repeated use of the system, contributing to cost reduction and improved process efficiency over time.

However, as previously discussed, substrate solubility remains a considerable limitation as it was not possible to dissolve 10 mM FUR and 50 mM MBA in the buffer system. To address this challenge, an alternative solvent system is required that enhances substrate solubility while preserving sufficient enzyme activity. Consequently, our research will focus on screening environmentally friendly (green) solvents that improve substrate solubility and, in turn, increase the STY of the process.

### Green Chemistry Metrics and Amine Donor Price per Reaction

The principles of green chemistry advocate minimizing excess substrate to avoid waste production during reactions. To identify the most environmentally friendly (“greenest”) reaction among those reported in the literature^1,7,8^ and our own proposed pathway using *N*-His_6_-ATA-wt, we evaluated the cost of various amine donors required to convert 10 mM FUR. For consistency, the comparison was based on a uniform FUR concentration of 10 mM, applying the amine donor:FUR ratios reported in each study, as summarized in Table 1. In addition, atom economy, excess reactant factor, and the reaction mass efficiency were calculated based on the substrate concentrations listed in Table 1.

**Table 1:** Green Chemistry Metrics for Batch FUR Biotransformations with Different Biocatalysts and Amine Donors and at Various FUR Concentrations Using Different Solvents^a^

amine donor	biocatalysts	reference	amine donor: acceptor molar ratio	FUR concentration, mM	solvent	yield [%]	reaction time [h]	atom economy [%]	excess reactant factor [-]	reaction mass efficiency [%]
IPA	(*S*)-ω-TAm from *Chromobacterium violaceum*	1	10:1	10	buffer	92	24	62.6	6.2	13.0
IPA	pEG 97-TA(*R*) from *Aspergillus terreus*	t.w.	10:1	10	buffer	40	24	62.6	6.2	5.7
ALA	*E. coli* NDTS cells with *At*AT from *Aspergillus terreus*	7	12:1	200	toluene/water	92	24	52.4	11.1	7.6
ALA	*E. coli* cells with TMEF (mutated *At*AT)	8	16:1	500	deep eutectic solvent	92	12	52.4	14.8	5.9
ALA	pEG 97-TA(*R*) from *Aspergillus terreus*	t.w.^b^	16:1	10	buffer	55	24	52.4	14.8	3.5
MBA	(*S*)-ω-TAm from *Chromobacterium violaceum*	1	5:1	5	buffer	80	12	44.7	6.3	11.1
MBA	*N*-His_6_-ATA-wt	t.w.	1:1	10	buffer	96	0.5	44.7	1.3	42.9

a To calculate the excess reactant factor and reaction mass efficiency, the FUR concentration and amine donor:acceptor molar ratio reported in the referenced studies were used.

b t.w. ...this work.

The cost of the amine donor was calculated assuming that a single experiment is performed in a batch reaction setup with a reactor volume of 5 mL and an initial FUR concentration of 10 mM. The use of ALA as an amine donor resulted in the highest cost due to its high price and the significant excess required to drive the reaction toward FA production. This increases costs and raises environmental concerns due to the large amount of amine donor required, which is reflected in the high excess reactant factor compared to the other reactions. In contrast, IPA was the most affordable amine donor, 106 times cheaper than ALA,^8^ even when used at a 10:1 ratio. However, the excessive reactant factor for this reaction is 4.88 times higher compared to our proposed reaction. In our proposed reaction, MBA is used as an amine donor in equimolar concentrations with FUR. Although our proposed reaction is slightly more expensive—only 0.0022 € more than the cheapest alternative—it shows significant improvements in the excess reactant factor, which was the lowest of all reactions considered.

In terms of atom economy, it is important to emphasize that the limitation of the formula is that it does not consider what excess reactant was used and what yield was obtained in the reaction. For example, comparing our proposed reaction with that of Dunbabin et al.,^1^ we use an equimolar MBA concentration, whereas their ratio was 5:1. Despite this difference, we obtained 16% higher FA yield, and according to the formula, both reactions had the same atom economy value (Table 1). In addition, our reaction had the lowest atom economy value compared to reactions using an even higher amine donor-to-acceptor ratio. When comparing our proposed reaction to the reaction with the highest amine donor-to-acceptor ratio, the atom economy improvement is almost 18%.

When considering the reaction mass efficiency, which takes into account the reactant excess in the reaction and the mass of the product obtained, our proposed pathway gives the highest value compared to other proposed reactions. Compared to other reactions (Table 1), it is 3.30 times more efficient than when IPA was used,^1^ 7.31 times more efficient for ALA,^8^ and 3.88 times more efficient when the MBA ratio is 5:1.^1^

Considering both the economic and environmental impacts, our proposed reaction with an equimolar concentration of FUR and MBA appears to provide a balanced approach that minimizes excess substrate consumption and associated waste while maintaining cost efficiency.

## Conclusions

In this study, a highly efficient and sustainable bioprocess for the synthesis of FA from FUR by biocatalysis in a microfluidic reactor was successfully developed. By screening different enzymes and amine donors at different molar ratios with FUR, a highly efficient biotransformation using *N*-His_6_-ATA-wt at a concentration of 0.4 mg/mL and MBA as amine donor at equimolar concentration with FUR was developed and an impressive yield of 96% was achieved within only 30 min. This approach eliminates the excessive need for amine donors previously required for all reported FUR bioamination reactions. This advancement not only lowers production costs but also conforms to green chemistry principles by minimizing waste generation.

Furthermore, the immobilization of the selected enzyme on synthesized, characterized, and functionalized MNPs, and their integration into a 3D-printed, magnetic field-assisted microreactor, demonstrated great potential for continuous FA production. The enzyme achieved a TTN of 2.04 × 10^7^, underlining its promising suitability for industrial-scale applications. This approach not only improves the efficiency and cost-effectiveness of FA production but also supports sustainable practices, reinforcing its potential for wider implementation in green chemistry initiatives. To support industrial implementation, future work will focus on optimizing the reaction medium using green solvents to enable industrially relevant substrate concentrations. In addition, model-based microreactor optimization including enzyme loading will be pursued to maximize process productivity.

## Supplementary Material



## Notes

It comprises HPLC chromatograms, results of immobilization conditions optimization, magnetization curves of MNPs, determination of reaction kinetic constants, enzyme deactivation rate constant, enzyme half-life estimation, and amine donor prices (PDF)

## Symbols and Abbreviations

ACP: acetophenone
ALA: d-alanine
ATA-wt: wild-type amine transaminase
*c*: molar concentration, mmol/L (mM)
FA: furfurylamine
FUR: furfural
GA: glutaraldehyde
IPA: isopropylamine
IPTG: isopropyl β-d-1-thiogalactopyranoside
*k*_cat_: catalytic constant, min^–1^ or day^–1^
*k*_d_: enzyme deactivation rate constant, day^–1^
LB: Luria–Bertani medium
MBA: (*S*)-(−)-α-methylbenzylamine
MNP: magnetite (Fe_3_O_4_) nanoparticle
PLP: pyridoxal-5′-phosphate
PTFE: polytetrafluoroethylene
PVP/HAP: poly­(*N*-vinyl-2-pyrrolidone)-capped Ru-supported hydroxyapatite
Schiff base 1: (*S*,*E*)-1-(furan-2-yl)-*N*-(1-phenylethyl)­methanimine
Schiff base 2: (*E*)-1-(furan-2-yl)-*N*-isopropylmethanimine
Schiff base 3: (*R*,*E*)-2-((furan-2-ylmethylene)­amino)­propanoic acid
Schiff base 4: (*E*)-1-(furan-2-yl)-*N*-(furan-2-ylmethyl)­methanimine
SEM: scanning electron microscope
STY: space-time yield, mol/(L h) or g/(L h)
*t*: time, min or h
*t*_1/2_: enzyme half-life, day
TA: transaminase
TEM: transmission electron microscopy
TTN: total turnover number
*V*: volume, mL or L
*v*_0_: initial rate, U/L
VSM: vibrating-sample magnetometer
γ: enzyme concentration, mg/mL
τ: residence time, min
ω-TA: ω-transaminase
