# Supplementary material for: Development of Sustainable Biocatalytic Furfurylamine Production in a Magnetic Field-Assisted Microfluidic Reactor
Source: ACS Sustain Chem Eng. 2025 Sep 11;13(38):15887–96. doi: 10.1021/acssuschemeng.5c04752 (PMC12486436; doi:10.1021/acssuschemeng.5c04752)
Supplement: Supplementary file 1 [file sc5c04752_si_001.pdf]

Supporting Information (SI)

Development of Sustainable Biocatalytic Furfurylamine Production in a Magnetic Field-Assisted Microfluidic Reactor

Marko Božinović[a], Marjan Jereb[a], Borut Šketa[a], Aljaž Gaber[a], Mojca Seručnik[a], Janez Košmrlj[a], Polona Žnidaršič-Plazl[a,*]

[a] University of Ljubljana, Faculty of Chemistry and Chemical Technology, Večna pot 113, 1000 Ljubljana, Slovenia

*Corresponding author:

Polona Žnidaršič-Plazl

E-mail: polona.znidarsic@fkkt.uni-lj.si

Tel.: +386 1 479 8572

Contents:

Total number of SI Pages: 11

Total number of Figures: 6

Total number of Tables: 3

Abbreviations:

[E]	enzyme concentration, mg/mL
ALA	D-alanine
FUR	furfural
GA	glutaraldehyde
IPA	isopropylamine
k_{cat}	catalytic constant, min^{-1} or day^{-1}
k_d	enzyme deactivation rate constant, day^{-1}
K_M	Michaelis constant, mM
MBA	(*S*)-(−)-α-methylbenzylamine
MNP	magnetite nanoparticle
R^2	correlation coefficient
Schiff base 1	(*S*,*E*)-1-(furan-2-yl)-*N*-(1-phenylethyl)methanimine
Schiff base 2	(*E*)-1-(furan-2-yl)-*N*-isopropylmethanimine
Schiff base 3	(*R*,*E*)-2-((furan-2-ylmethylene)amino)propanoic acid
Schiff base 4	(*E*)-1-(furan-2-yl)-*N*-(furan-2-ylmethyl)methanimine
t	time, min or h
$t_{1/2}$	enzyme half-life, day
TTN	total turnover number
γ	enzyme concentration, mg/mL
ω-TA	transaminase

By-product formation during non-enzymatic and enzymatic reaction

After mixing equimolar concentrations of the tested amine donors (D-alanine (ALA), isopropylamine (IPA), (*S*)-(−)-α-methylbenzylamine (MBA)) with furfural (FUR) and furfurylamine (FA) dissolved in potassium phosphate buffer (pH 7.5, 100 mM), and incubation at 30°C and stirred with a magnetic stirrer at 300 rpm without enzyme addition for 5 min, the reaction mixtures were analyzed by ^{1}H NMR spectroscopy (available at the Repository data file).

Based on the results of non-enzymatic reactions obtained by ^{1}H NMR analysis, the reaction scheme shown in Figure S1 was proposed. As evident, FUR reacts spontaneously with all tested amine donors and FA, leading to the formation of Schiff bases ((*S*,*E*)-1-(furan-2-yl)-*N*-(1-phenylethyl)methanimine (Schiff base 1), (*E*)-1-(furan-2-yl)-*N*-isopropylmethanimine (Schiff base 2), (*R*,*E*)-2-((furan-2-ylmethylene)amino)propanoic acid (Schiff base 3), and (*E*)-1-(furan-2-yl)-*N*-(furan-2-ylmethyl)methanimine (Schiff base 4)).

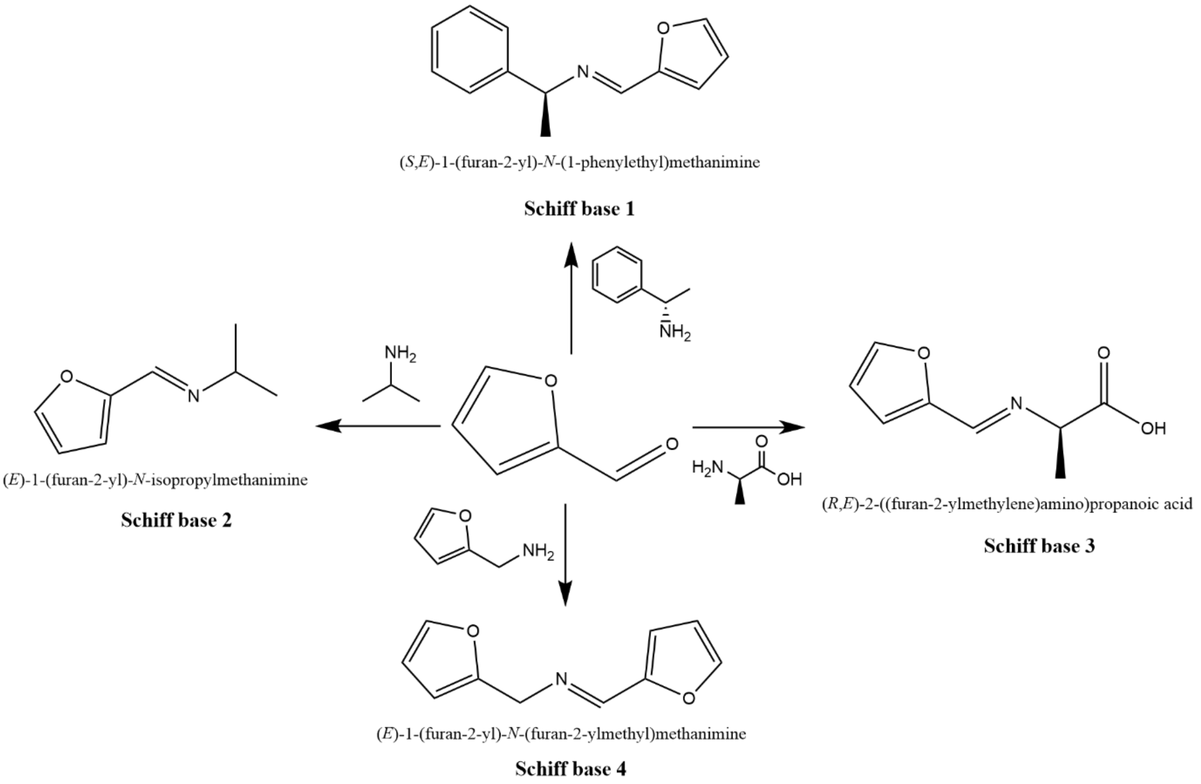

Figure S1. The formation of Schiff bases by non-enzymatic reactions of furfural (FUR) with various amine donors and with furfurylamine (FA) as confirmed by ^{1}H NMR spectra (available at the Repository data file).

The enzymatic reaction using ω-transaminase (ω-TA) pEG 97-TA(*R*)-AspTerr with 10 mM FUR and IPA, ALA, and MBA was further investigated using an equimolar donor-to-acceptor ratio. After incubation for 5 min, the reactions were quenched by 5-fold dilution with potassium phosphate buffer (100 mM, pH 7.5) preheated to 100°C, and the mixture was heated to 100°C for another 5 min. As shown in Figure S2, HPLC analysis confirmed the formation of Schiff bases 1-4 also during the enzymatic reaction, depending on the amine donor used. The retention times of Schiff bases 1, 2, 3, and 4 were 4.852, 5.562, 6.492, and 6.524 min, respectively.

a)

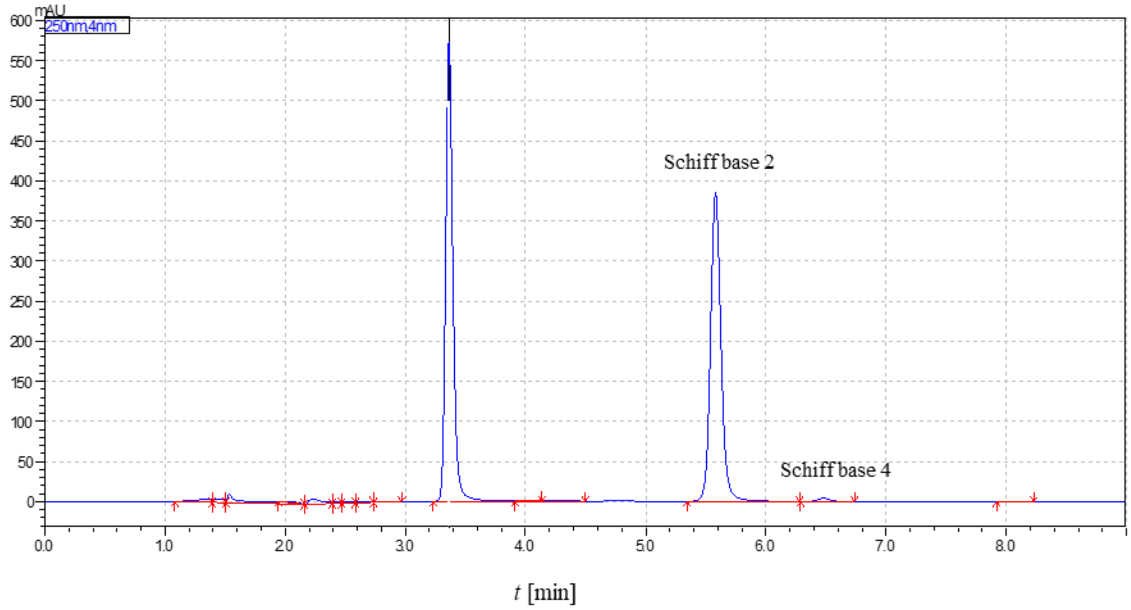

b)

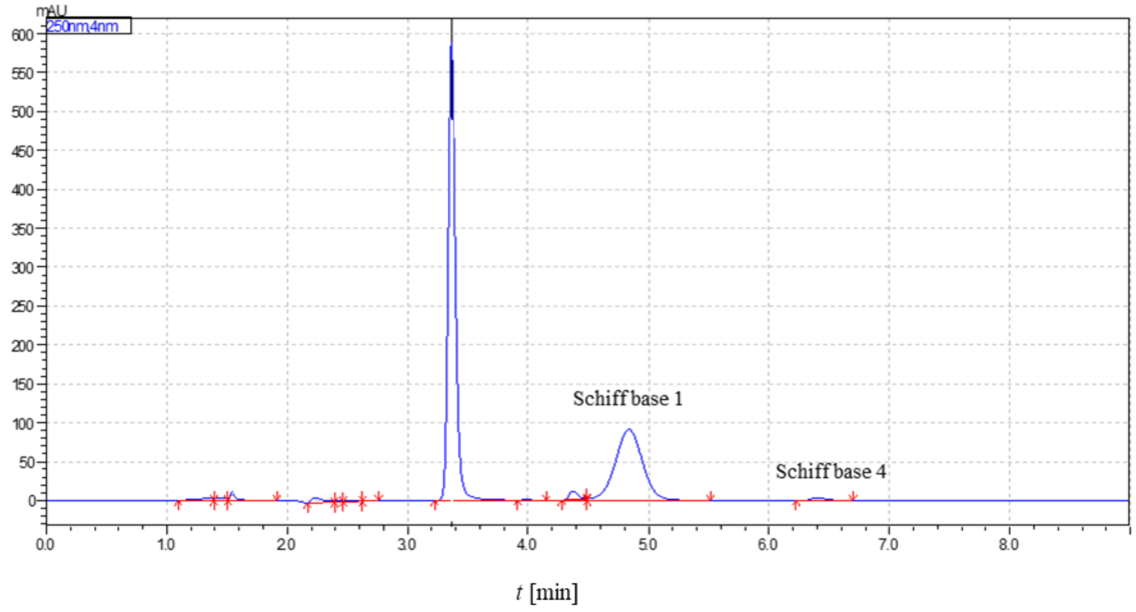

c)

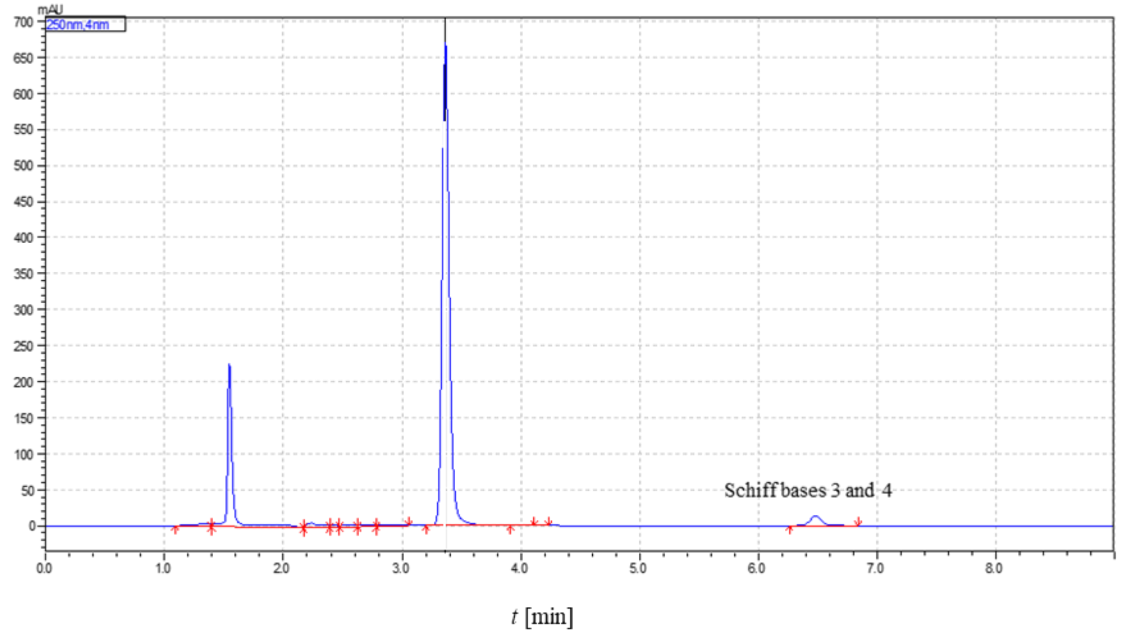

Figure S2. HPLC chromatograms obtained after 5 min of the reaction between (a) IPA and FUR, (b) MBA and FUR and c) ALA and FUR, with the enzyme pEG 97-TA(*R*)-AspTerr at equimolar concentration of amine donor and acceptor (10 mM); enzyme concentration (γ) 0.1 mg/mL, pH 7.5 and 30°C.

To determine whether a higher amine donor-to-acceptor ratio shifts the reaction toward FA formation, the enzymatic reaction was further tested using pEG 97-TA(*R*)-AspTerr and FUR with IPA and ALA. As shown in Figure S3, HPLC analysis also confirmed the formation of Schiff bases 2-4.

a)

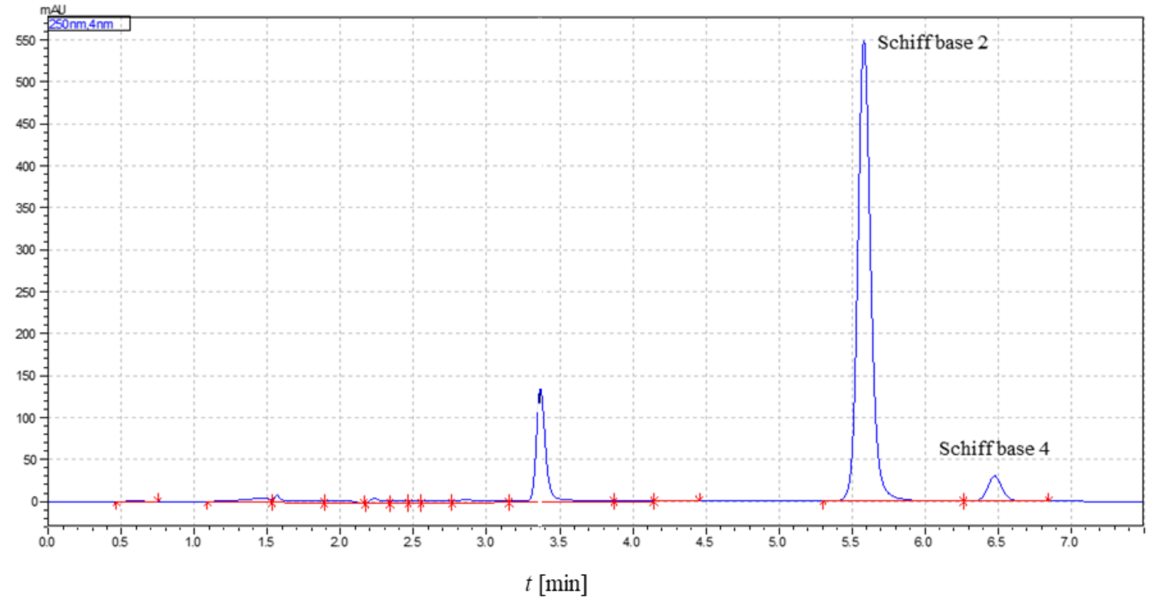

b)

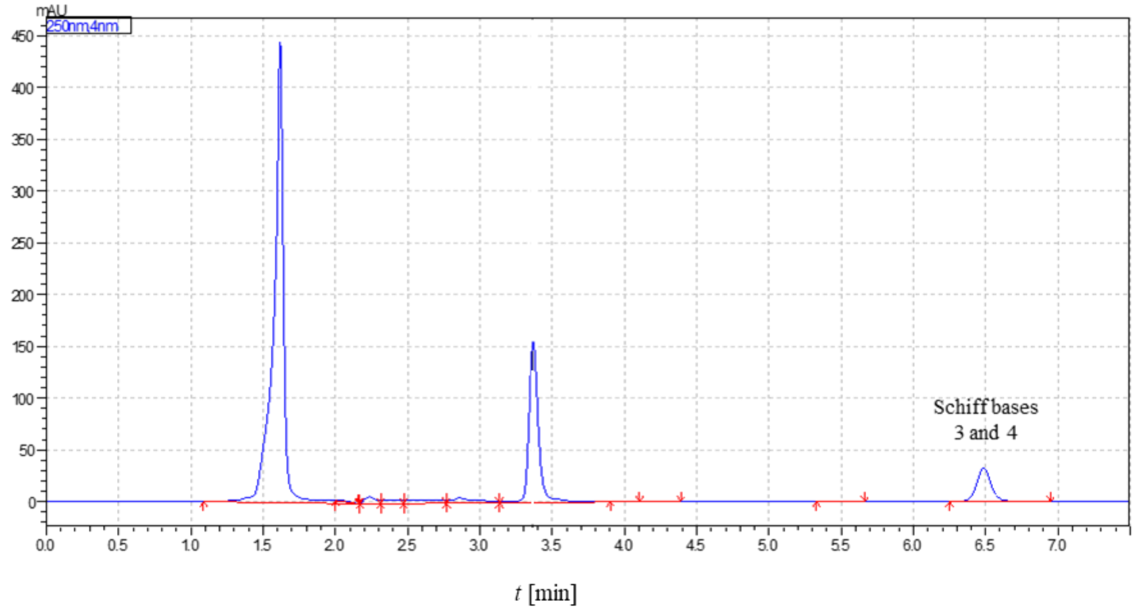

Figure S3. HPLC chromatograms obtained after 3 h of reaction between (a) IPA and FUR at a 10:1 molar ratio, and (b) ALA and FUR at a 16:1 molar ratio, catalyzed by pEG 97-TA(*R*)-AspTerr (γ=0.1 mg/mL) at pH 7.5 and 30 °C.

Characterization of synthesized magnetite nanoparticles

Magnetic characterization was performed at room temperature using a vibrating-sample magnetometer. The magnetite nanoparticles (MNPs) exhibited ferrimagnetic behavior, as evidenced by the hysteresis loop shown in Figure S4.

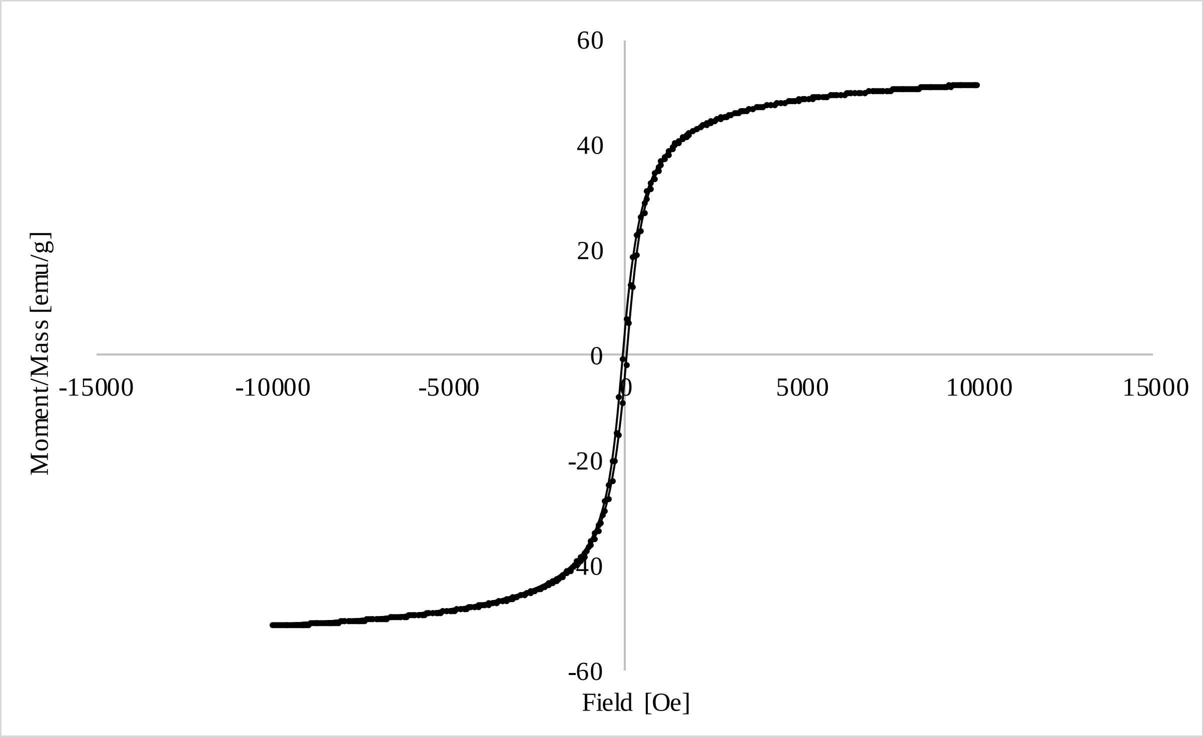

Figure S4. Magnetization curves of synthesized MNPs.

Optimization of *N*-His$_6$-ATA-wt batch immobilization on MNPs

The effect of enzyme to MNP mass ratio on *N*-His$_6$-ATA-wt immobilization yield and recovered activity was preliminarily evaluated in a batch process at fixed GA concentration (Table S1).

Table S1. The calculated immobilization yield and recovered activity of *N*-His$_6$-ATA-wt at various enzyme-to-MNPs mass ratio and at 2 % (v/v) GA concentration. Standard deviation is given for triplicate samples.

Enzyme amount per dry support [mg/g]	Immobilization yield [%]	Recovered activity [%]
80	88.71±0.25	92.80±2.58
100	80.68±0.24	90.38±1.59
120	76.54±0.18	84.25±0.72

Since 80 mg enzyme/g dry MNPs gave the best results in terms of both immobilization yield and recovered activity, this condition was used to evaluate the effect of glutaraldehyde (GA) concentration on both parameters (Table S2).

Table S2. The calculated immobilization yield and recovered activity of *N*-His$_6$-ATA-wt at various GA concentrations and at 80 mg/g enzyme-to-MNPs mass ratio.

GA concentration [%] (v/v)	Immobilization yield [%]	Recovered activity [%]
1	92.36±0.91	65.88±1.30
2	88.71±0.25	92.80±2.58
3	75.38±0.66	82.32±0.58

As can be seen from Table S2, 2% (v/v) GA gave the best recovered activity, so this condition was used in further experiments.

Determination of enzyme kinetic constants

To calculate the total turnover number (TTN) for *N*-His_6-ATA-wt, the catalyst constant (k_{cat}), was first estimated using the Ping-Pong Bi-Bi kinetic model without inhibition (eq S1):

$$r = \frac{k_{cat} \cdot [E] \cdot [MBA] \cdot [FUR]}{K_{M\_FUR} \cdot [MBA] + K_{M\_MBA} \cdot [FUR] + [FUR] \cdot [MBA]} \quad (S1)$$

Here, K_{M_FUR} and K_{M_MBA} are the Michaelis constants for FUR and MBA, respectively, while [MBA], [FUR] and [E] represent the concentrations of MBA, FUR, and enzyme. Experimental data were obtained from a batch biotransformation reaction conducted with equimolar concentrations of FUR and MBA (10 mM each) and an enzyme concentration of 0.4 mg/mL. All other conditions were identical to those described in the *Experimental Section* under *Furfural biotransformation and evaluation of enzyme specific activities.*

The data presented in Figure S5 were fitted using the kinetic model eq S1, assuming equal K_{M_MBA} and K_{M_FUR} values of 7.9 mM. Parameter k_{cat} estimation was performed using the least squares method, and simulation was carried out in Mathematica 13.3 (Wolfram Research, Champaign, IL, USA).

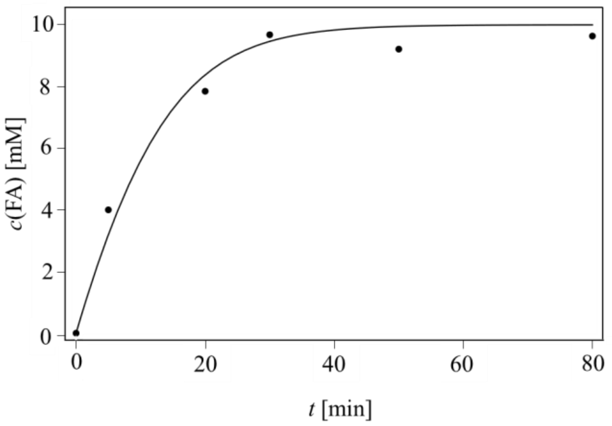

Figure S5. Experimental data (dots) showing the time-dependent concentration of FA from batch biotransformation with initial concentrations of 10 mM FUR and MBA, and *N*-His_6-ATA-wt at γ=0.4 mg/mL. Model simulations (solid line) of FA concentrations were calculated using eq. S1, assuming K_M value of 7.9 mM for both FUR and MBA, and a k_{cat} of 13 s^{-1}.

Based on the model fit, the catalytic constant k_{cat} was calculated to be 13 s^{-1} (783.46 min^{-1}). As shown in Figure S5, the model using the selected parameters exhibited good agreement with the experimental data.

Determination of enzyme deactivation rate constant and enzyme half-life

A deactivation rate constant and enzyme half-life were assessed for *N*-His$_6$-ATA-wt based on data obtained from the operational stability of a continuously operated microreactor for using immobilized *N*-His$_6$-ATA-wt on MNPs and employing MBA as the amine donor at an equimolar concentration with FUR (10 mM), at pH 7.5 and room temperature. By plotting *ln*(observed activity) against time (t), and assuming first-order kinetics (eq S2), the enzyme deactivation rate constant (k_d) was determined from the slope of the resulting linear regression. The correlation coefficient (R^2) is also calculated as a statistical measure of how well the regression line fits the data. The observed activity was determined as described in *Experimental Section*. The results are shown in Figure S6.

$$ln(\text{observed activity}) = ln(\text{observed activity})_{\text{day 0}} - k_d t \qquad \text{(S2)}$$

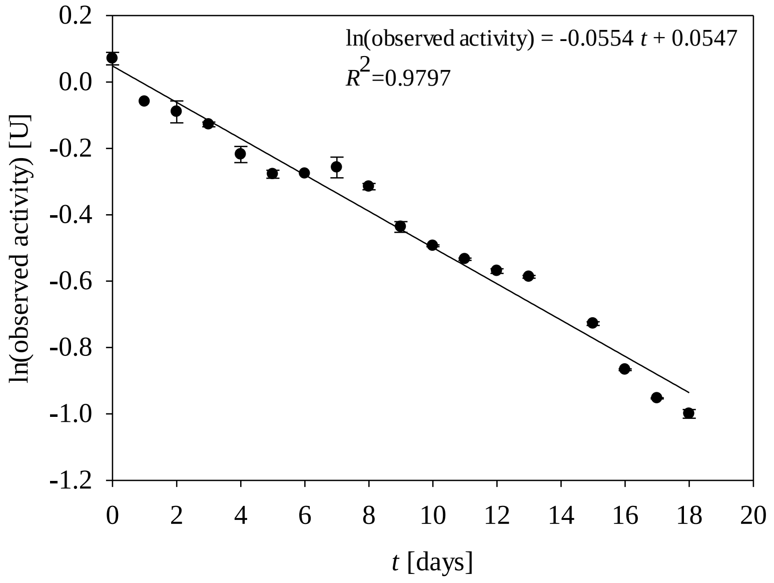

Figure S6. Experimental data (dots) from a continuously operated microreactor using *N*-His$_6$-ATA-wt immobilized on MNPs with MBA at an equimolar concentration (10 mM) to FUR, conducted at pH 7.5 and room temperature. Error bars represent the standard deviation from triplicate experiments. The solid line shows the linear fit based on eq S2, and the corresponding linear equation and R^2 value are indicated.

The enzyme half-life ($t_{1/2}$) was calculated using eq S3 and was 0.0554 days^{-1}.

$$t_{1/2} = \frac{ln2}{k_d} \qquad \text{(S3)}$$

Cost-effectiveness estimation of FUR amination

To determine the cost-effectiveness of FUR amination reactions, the prices of the tested amine donors were taken from the catalogue of Merck KGaA, Darmstadt, Germany (Table S3).

Table S3. The prices of amine donors from Merck KGaA catalogue as of May 7th, 2025.

**Chemical**	**Quantity**	**Price [€]**
IPA	100 mL	26.60
ALA	5 g	84.80
MBA	25 g	56.20
